# Anti-Inflammatory Effect of Specialized Proresolving Lipid Mediators on Mesenchymal Stem Cells: An In Vitro Study

**DOI:** 10.3390/cells12010122

**Published:** 2022-12-28

**Authors:** Shahd AlZahrani, Zakia Shinwari, Ameera Gaafar, Ayodele Alaiya, Ahmed Al-Kahtani

**Affiliations:** 1 Department of Restorative Dental Sciences, College of Dentistry, King Saud University, Riyadh 11612, Saudi Arabia; 2 Therapeutics & Biomarker Discovery for Clinical Applications, Stem Cell & Tissue Re-Engineering Program, King Faisal Specialist Hospital and Research Centre, P.O. Box 3354, Riyadh 11211, Saudi Arabia

**Keywords:** hBMMSCs, SPMs, Resolvin E1, Maresin1, stem cells, inflammation, LPS

## Abstract

An interconnection between tissue inflammation and regeneration has been established through the regulation of defense and repair mechanisms within diseased dental tissue triggered by the release of immune-resolvent mediators. To better our understanding of the role of specific pro-resolving mediators (SPMs) in inflamed human bone marrow-derived mesenchymal stem cells (hBMMSCs), we studied the effects of Resolvin E1 (RvE1) and Maresin 1 (MaR1) in lipopoly-saccharide (LPS) stimulated hBMMSCs. The hBMMSCs were divided into five different groups, each of which was treated with or without SPMs. Group-1: negative control (no LPS stimulation), Group-2: positive control (LPS-stimulated), Group-3: RvE1 100 nM + 1 μg/mL LPS, Group-4: MaR1 100 nM + 1 µg/mL LPS, and Group-5: RvE1 100 nM + MaR1100 nM + 1 μg/mL LPS. Cell proliferation, apoptosis, migration, colony formation, Western blotting, cytokine array, and LC/MS analysis were all performed on each group to determine the impact of SPMs on inflammatory stem cells. According to our data, RvE1 plus MaR1 effectively reduced inflammation in hBMMSCs. In particular, IL-4, 1L-10, and TGF-β1 activation and downregulation of RANKL, TNF-α, and IFN-γ compared to groups receiving single SPM were shown to be significantly different (Group 3 and 4). In addition, the LC/MS analysis revealed the differentially regulated peptide’s role in immunological pathways that define the cellular state against inflammation. Inflamed hBMMSCs treated with a combination of Resolvin E1 (RvE1) and Maresin 1 (MaR1) promoted the highest inflammatory resolution compared to the other groups; this finding suggests a potential new approach of treating bacterially induced dental infections.

## 1. Introduction

Dental-pulp tissue may undergo rapid degeneration and necrosis when exposed to external factors such as microbial infection, mechanical/chemical irritation, surgical interventions, and tissue injury which will eventually lead to apical periodontitis if left untreated [1,2]. A polymicrobial infection of pulpal origin is the primary cause of the inflammatory condition known as apical periodontitis [3]. The infectious endodontic microbiota is dominated by gram-negative bacterial species. These bacteria have the capacity to multiply and degrade apically, releasing lipopolysaccharide (LPS), which can cause and prolong apical periodontitis [4]. By altering the tissue’s defense and repair mechanisms, which are sparked by the release of growth factors, neuropeptides, cytokines, and chemokines, an important interrelationship between inflammation and tissue regeneration is emerging today [5]. A genus of biosynthesized specialized pro-resolving mediators (SPMs) regulates the onset, duration, and resolution of inflammation by mediating the activation and clearance of anti- and pro-inflammatory signals which limit inflammation’s destructive effects on diseased tissues [6].

SPMs, such as Lipoxin (LX), Resolvin E (RvE), Maresin (MaR), and Protectin (PD), are derived from the metabolism of lipid components such as docosapentaenoic acid (DPA), eicosapentaenoic acid (EPA), docosahexaenoic acid (DHA), omega-3 polyunsaturated fatty acids, and arachidonic acid (AA). SPMs are stereoselective, and the majority of them have complete stereochemical assignments. These SPMs are small lipid molecules with strong biological activity that has been demonstrated under experimental conditions [7]. Since the biological function of these pro-resolving inflammatory mediators is to reduce the negative effects of insulting influxes and to promote the formation of reparative tissue by differentiating T cells into a reparative stage, their combined use may enhance cell proliferation, migration, survival ability, and immunomodulation to a greater extent than treatment with one mediator alone. Particularly in oral/dental pathology, analogues of lipoxin A4 (LXA4) have been shown to reduce recruitment of neutrophil at the infection site of P. gingivalis infection [8]. In an animal model, leukocyte infiltration into an inflammation site was reduced on treatment with LXA4, the enzyme producing lipoxins [9], which highlights the role of lipoxins in chronic infection. Whereas the pre-eminent role of MaR1 is anti-inflammatory, in addition to that, it also promotes tissue regeneration [10], regulates cell autophagy, cytokine release, and helps in survival of human periodontal ligament (PDL) [11]. Likewise, resolvins (RvE1) play a part in fostering defense against bacterial periodontitis. RvE1 reduces cytokines and superoxide production [12,13], infiltration of neutrophil [13], and macrophage activity [14] in localized aggressive periodontitis (LAP). Topical RvE1 therapy reduces localized and systemic inflammation in animal models of LAP and enables the host to replenish the bone mass and the lost tissue [14]. In addition, RvE1 has the ability to restore phagocyte activity, contributing to re-establishing the tissue homeostasis. RvE1 and MaR1 were selected in our study because both of them are derived from omega-3 polyunsaturated fatty acid, and both have the potential to stimulate pro-regenerative activities, regulate wound healing, and reverse tissue destruction.

However, to the best of our knowledge, no research has been conducted to determine whether Resolvin E1 (RvE1) and Maresin 1 (MaR1) alone or in combination is an effective treatment for inflammatory lesions involving human bone marrow-derived mesenchymal stem cells (hBMMSCs). The purpose of this in vitro research was to compare the effects of Resolvin E1 (RvE1) and Maresin 1 (MaR1) on human bone marrow-derived mesenchymal stem cell (hBMMSC) cellular viability, proliferation, migration, survival capacity, and expression of inflammation-related cytokines in an inflammatory microenvironment. The development of a therapy for preserving stem cell vitality will benefit from a deeper assessment of the role that RvE1 and MaR1 play in this process and will aid in the development of a therapy for preserving stem cell vitality.

## 2. Materials and Methods

### 2.1. Preparation of the Experiment

#### 2.1.1. Cell Culture

Immortalized human bone marrow mesenchymal stem cells (hBMMSC) were obtained from the Stem Cell Laboratory, Medical College of King Saud University, King Khaled University Hospital, Riyadh, Saudi Arabia. The cells were cultured in DMEM-high glucose medium ((Sigma-Aldrich), St. Louis, MO, USA) supplemented with 10% FBS (Gibco, Carlsbad, Los Angeles, CA, USA), 4 mM L-glutamine (Gibco, Carlsbad, Los Angeles, CA, USA), and 1 mM sodium pyruvate (Sigma-Aldrich), St. Louis, MO, USA and incubated at 37 °C with 5% CO_2_. Confluent cells were harvested using 0.25% trypsin-EDTA (Gibco, Carlsbad, Los Angeles, CA, USA) and washed in 1X PBS (Sigma-Aldrich, St. Louis, MO, USA) and used in subsequent experiments.

#### 2.1.2. Characterization

The International Society of Cellular Therapy (ISCT) recommendation was followed for characterization of hBMMSCs. Harvested cells were stained for specific surface markers using the Human MSC Analysis Kit (BD Biosciences, Franklin Lakes, NJ, USA). Our cells stained positively for CD73, CD44, CD105, and CD90 and negatively for hematopoietic markers CD133, CD14, MHC-II, CD45, and CD34. The data were generated using a BD LSR Flow Cytometer and analyzed using BD FACSDiva Software (BD Biosciences, Franklin Lakes, NJ, USA).

#### 2.1.3. Assessment of Inflammation-Related Protein Expression in Response to LPS Treatment by Western Blot Analysis

Lipopolysaccharide (LPS) treatment was selected as a stimulus to provide the theoretical basis for the establishment of an LPS-induced inflammation model. LPS powder (Sigma-Aldrich), St. Louis, MO, USA) was dissolved in Hank’s Balanced Salt solution without Ca^2+^ and Mg^2+^ (Thermo Scientific, Rockford, IL, USA) to obtain a stock solution of 1 mg/mL LPS and stored at −20 °C for future use. Cells were stimulated with 1 µg/mL LPS for 24 h and the protein expression of TNF-α (sc-52746) (Santa Cruz Biotechnology, Inc., Dallas, TX, USA) was assessed by Western blot to confirm the inflammatory induction of LPS treatment on hBMMSCs.

#### 2.1.4. Drug Preparation and Optimization

Resolvin E1 (RvE1) and Maresin1 (MaR1) specialized synthetic pro-resolving inflammatory mediators were commercially purchased from GLPBIO Technology, Montclair, CA, USA. Stock solutions (10 µM) of each were prepared in absolute ethanol and stored at −80 °C for further use. Real-Time Cell Analyzer (RTCA) (ACEA Biosciences, Santa Clara, CA, USA) was used to investigate the proliferation and migration rates under different concentrations (10, 50, 100, 150 nM) of both mediators. In addition, to assess the apoptotic effect of these agents, treated cells were stained with annexin V/PI using Alexa Fluro 488 Annexin V/Dead Cell Apoptosis Kit (Molecular Probes, Life Technologies, Eugene, Ore, USA) and analyzed using BD LSR Flow Cytometer and BD FACSDiva Software (BD Biosciences, Franklin Lakes, NJ, USA). The selected doses were based on previously reported dose–response studies [15,16] with slight modifications according to our experimental optimization. Thereafter, 100 nM concentration was selected for both mediators RvE1 and MaR1 for further experiments.

### 2.2. Design of the Experiment

hBMMSCs were seeded in three 6-well plates at a density of 8 × 10^5^/well. Upon confluence (80–90%), the wells were arranged into five treatment groups. Group-1: Negative control (unstimulated/no LPS), Group-2: positive control (LPS stimulated), Group-3: RvE1 100 nM + 1 µg/mL LPS, Group-4: MaR1 100 nM + 1 µg/mL LPS, and Group-5: RvE1 100 nM + MaR1 100 nM + 1 µg/mL LPS. Cells of the same passage were used for each experiment and incubated at 37 °C with 5% CO_2_. All experiments were carried out three times, except for the Western blot that was repeated two times, to confirm the findings. As a part of the procedure, extended treatments required media changing two to three times per week throughout the experimental work.

### 2.3. Cell Proliferation and Viability Assay

Real-time cell analyzer (RTCA) was used to measure cell proliferation and viability. This system offers a superior substitute for the MTT and BrdU cell proliferation and viability assays as a quantitative platform that measures cellular parameters, including, number of living cells, cell proliferation rate, size and shape of the cells, and cell adhesion strength. hBMMSCs were seeded in an E-16 plate at a density of 5 × 10^3^/well and incubated with 1 μg/mL of LPS for 24 h, with the exception of the negative control group, which only received culture medium. After 24 h, cells of the experimental groups were treated with 100 nM of RvE1, 100 nM of MaR1, or mixture of both drugs with a final concentration of 200 nM. Proliferation of treated hBMMSCs was continuously monitored and the cell index measured over a 7-day period. This experiment was conducted three times (*n* = 3) to confirm the findings.

### 2.4. Cell Apoptosis Assay

hBMMSCs were cultured in high glucose DMEM medium with supplements using 6-well plates. As the cells completed 60% confluence, they were grouped and treated for 72 h with 100 nM RvE1 + 1 μg/mL LPS, 100 nM MaR1+ 1 μg/mL LPS, and a combination of 100 nM RvE1 + 100 nM MaR1 + 1 μg/mL of LPS. Detached and adherent cells were harvested, centrifuged, and then stained with propidium iodide (PI) and Alexa Fluro 488 Annexin V using Alexa Fluro 488 Annexin V/Dead Cell Apoptosis Kit (Molecular Probes, Life Technologies, Eugene, Ore, USA). Stained cells were then analyzed by flow cytometry and BD FACSDiva Software (BD Biosciences, Franklin Lakes, NJ, USA). The experiment was carried out three times (*n* = 3) to confirm the findings.

### 2.5. Migration Assay

We also used the RTCA to track the kinetics of cell migration. Using the CIM-plate 16, migration was evaluated in accordance with the manufacturer’s recommendations. Briefly, the lower chamber wells were filled with 120 µL media containing 10% FBS. The upper chamber was placed onto the lower chamber with the sensor surface facing down using CIM-Plate 16 Assembly Tool. Only 25,000 cells (hBMMSCs) were prepared in 75 µL serum free medium and loaded onto the upper chamber and kept for 30 min in the incubator. The cells were not subjected to starvation for any particular period of time, as these cells could not tolerate starvation. The treatments of desire (same experimental groups above) were prepared in another 75 µL serum free medium and added to the cells. The plates were placed into the RTCA for 24 h incubation and all samples underwent quadruple runs. Cell indices were measured, and migration was continuously observed using the RTCA instrument software.

### 2.6. Colony-Forming Assay

The colony forming capability of the treated cells were evaluated as previously described [17]. Briefly, hBMMSCs were seeded in four 6-well plates at a density of 500 cells/well to test the ability of a single cell to survive and develop into colonies. Cells were incubated at 37 °C for another two days before adding the desired treatments (same experimental groups above) according to each assigned group. After 14 days of seeding date, plates were fixed with 3.7% formaldehyde (in 1X PBS) to each well, then stained with crystal violet stain; aggregates of 50 cells or more were scored as 1 colony-forming unit (CFU). Plating efficiency (PE) was calculated for each well in all experimental groups which were repeated three times (*n* = 3). PE = (number of colonies formed/number of cells seeded) × 100%.

### 2.7. Western Blot

Cells were cultured for 24 h in a set of 10 T100 petri dishes, each plate containing 1 × 10^6^ hBMMSCs in the presence of 1 μg/mL LPS. The cells were then grouped (two plates/group) and treated as follows: Group-1: Negative control (unstimulated/no LPS), Group-2: positive control (LPS stimulated), Group-3: RvE1 100 nM + 1 µg/mL LPS. Others are Group-4: MaR1 100 nM + 1 µg/mL LPS, and Group-5: RvE1 100 nM + MaR1 100 nM + 1 µg/mL LPS with RvE1 and MaR1 for 7 days. Thereafter, cells were lysed using RapiGest buffer, (Waters Corporation, Milford, CT, USA) and proteins were extracted for Western blot analysis.

Protein concentration was measured using the Bradford method and 30 µg of protein was loaded onto SDS-PAGE before being transferred to PVDF membranes. The membranes were incubated at 4 °C overnight with primary antibodies (dilution 1:1000), TNF-α (SC-52746), and receptor activator of nuclear factor kappa-Β ligand (RANKL) (SC-377079), Interferon-γ (IFN- γ) (SC-373727). Interleukin-10 (IL-10) (SC-8438), transforming growth factor-β1 (TGF-β1) (SC-130348) (purchased from Santa Cruz Biotechnology Inc., Dallas, TX, USA), Interleukin-4 (IL-4) (MAB204) (purchased from R&D systems Bio-techne, Minneapolis, MN, USA) and GAPDH used as an internal control, and inter ECL detection reagents were used to examine the membranes (Pierce; Thermo Fisher Scientific, Inc., Waltham, Mass., USA). iBright Imaging Systems was utilized to visualize the images. The PDQuest^TM^ Software was used to perform band quantifications (version 7.3.1; Bio-Rad Laboratories, Hercules, CA, USA). This experiment was conducted two times (*n =* 2) to confirm the findings.

### 2.8. Human Cytokine Antibody Array C2000

RayBio^®^ AAH-CYT-6 array membrane (Raybiotech, Peachtree Corners, GA, USA) designed for semi-quantitative detection of 60 human proteins, was used to compare selected cytokine expression in the supernatants of experimental groups according to the manufacturer’s instructions. Briefly, the array membrane was incubated at 25 °C overnight with the blocking buffer in each well. The membranes were washed with wash buffer I and then with wash buffer II for 5 min at 25 °C. The membranes were incubated with biotinylated antibody cocktail overnight at 4 °C, thereafter, washed before being incubated with 1X HRP-Streptavidin at 4 °C overnight. Images were created using two different exposure times after the membranes were transferred to the chemiluminescence imaging system (2 s and 30 s). The analysis was carried out on experimental groups only due to the limited supply of membranes (*n* = 3).

### 2.9. Protein in Solution-Digestion

All samples were subjected to in-solution protein digestion prior to label-free LC–MS/MS analysis, as previously described. The hBMMSCs from the five sample groups were lysed and equal amounts of proteins (100 µg) from Group 1 to 5 were subjected to tryptic digestion as previously described [18]. Briefly, sample proteins were denatured with RapiGest SF (Waters, Manchester, UK) for 15 min at 80 °C and then 10 mM DTT was added at 60 °C, and then alkylated in the dark at room temperature. The samples were then trypsinized with overnight incubation at 37 °C and the reaction quenched with 37% hydrochloric acid for 15 min, followed by centrifugation at 13,000 rpm for 10 min. Prior to liquid chromatography/mass spectroscopic analysis, the samples were diluted in 0.1% formic acid and spiked with alcohol dehydrogenase for absolute quantification.

#### 2.9.1. Protein Characterization by Label-Free Liquid Chromatography/Mass Spectrometry

Synapt G2 HDMS instrument, 1D Nano Acquity liquid chromatography mass spectrometry was used to analyze each sample (Waters, Manchester, UK). The MassLynx tune page was used to optimize the instrument settings before analysis, as previously described [17,18]. Briefly, leucine enkephalin (2 ng/L) and 500 fmol [Glu]1-fibrinopeptide B were used, by the Mass Lynx IntelliStart for detector setup and mass calibration respectively. A 3 Kv capillary voltage, 50 V and 5 V sample cones and extraction cones, and 80 °C source temperature were also set in the tune page parameters. Positive ion (Trizaic Nano Source) mode nanoESI was used for all analyses. The AcquityTM HSS T3 85 um × 100 mm Trizaic Nano tile column was loaded with equal amounts (3 µg) of protein digest per sample. All samples were subjected to a flow rate of 0.450 µL/min using the mobile phases A1 with 99% (water + 0.1% formic acid) and B1 with 1% (acetonitrile + 0.1% formic acid). The data were acquired between 50 to 2000 Da with a total acquisition time of 120 min as previously described [17,18] using the Mass Lynx version 4.1.

#### 2.9.2. Data Analysis

The data were processed using Progenesis QI V4 as previously described [17,18]. The Uniprot non-redundant human protein sequence database was queried using the acquired list of peptide ions for protein identification (www.uniprot.org, accessed on 10 May 2022). The generated data were filtered utilizing multivariate statistical analyses and protein expression changes with *p* value less than 0.05 while a fold change of at least 2-fold and greater was considered to be statistically significant.

#### 2.9.3. Statistical Analysis

Data analysis was performed using SPSS version 26.0 software (IBM Inc., Armonk, NY, USA). Descriptive statistics were used to describe the quantitative outcome variables (migration, proliferation, CFA, Western blot, and human cytokine array). Repeated measures analysis of variance were followed by Tukey’s multiple comparison test for different time points analysis. One-way analysis of variance with Tukey’s multiple comparison test was used to compare (i) the mean values of CFA, (ii) the mean values of Western blot among the five study groups, and (iii) the mean values of human cytokine array among the three study groups (3, 4, and 5). *p*-value ≤ 0.05 was considered as significant.

## 3. Results

### 3.1. Cell Proliferation and Viability Assay

**Figure 1:**
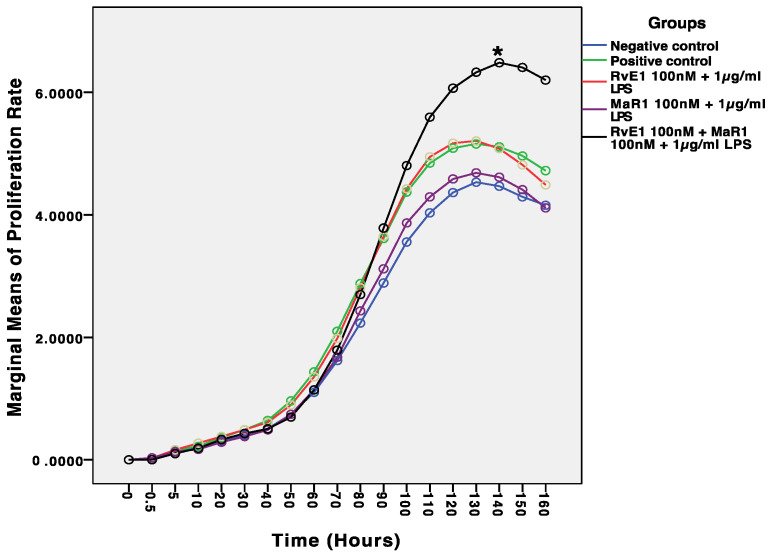
Comparison of mean proliferation values among the five study groups across different time points. The proliferation and viability assays experiments were repeated three times (*n* = 3) and the average values used to confirm the findings. The proliferation rate of * Group-5 (RvE1 + MaR1 + LPS) was significantly higher than the other four groups (*p* ≤ 0.0001).

The comparison of the mean values of proliferation across 18 time points revealed a statistically significant difference between the five study groups (F = 939.72, *p* ≤ 0.0001). In addition, the interaction term (Groups * Time points) is statistically different (F = 5.35, *p* ≤ 0.0001). Among the five groups, the mean proliferation rate of Group-5 is statistically significantly higher than the mean proliferation rate of the other studied groups across eight time points (from 90 h to 160 h) (Figure 1). Whereas no statistically significant difference was observed among the four study groups (Group-1: Negative control, Group-2: Positive control, Group-3: RvE1 100 nM +1 μg/mL LPS, Group-4: MaR1 100 nM +1 μg/mL LPS).

### 3.2. Cell Apoptosis Assay

**Figure 2:**
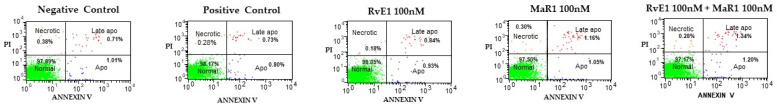
Rates of apoptosis in hBMMSCs in the control and experimental groups after RvE1 and MaR1 treatment alone or together in response to LPS-induced inflammation using Annexin V/propidium iodide double staining by flow cytometry. The experiments were repeated three times (*n* = 3) and there were no significant differences in apoptosis between the five study groups (*p* = 0.71). Representative graph shows the fractions of viable and dead cells across the different study groups.

In order to determine the roles of RvE1 and MaR1 in harm-induced apoptosis, the percentage of cell death (early + late apoptosis) in hBMMSCs was measured across all of the different groups using flow cytometry. Apoptosis rates were 1.72%, 1.53%, 1.77%, 2.21%, and 2.54% in the negative control, positive control, RvE1 100 nM + 1 g/mL LPS, MaR1 100 nM 1 g/mL LPS, and RvE1 + MaR1 100 nM + 1 g/mL LPS groups, respectively. The rates of apoptosis did not differ between the study groups, suggesting that the presence of SPMs did not influence cell death (F = 0.55, *p* = 0.71) (Figure 2).

### 3.3. Migration Assay

**Figure 3:**
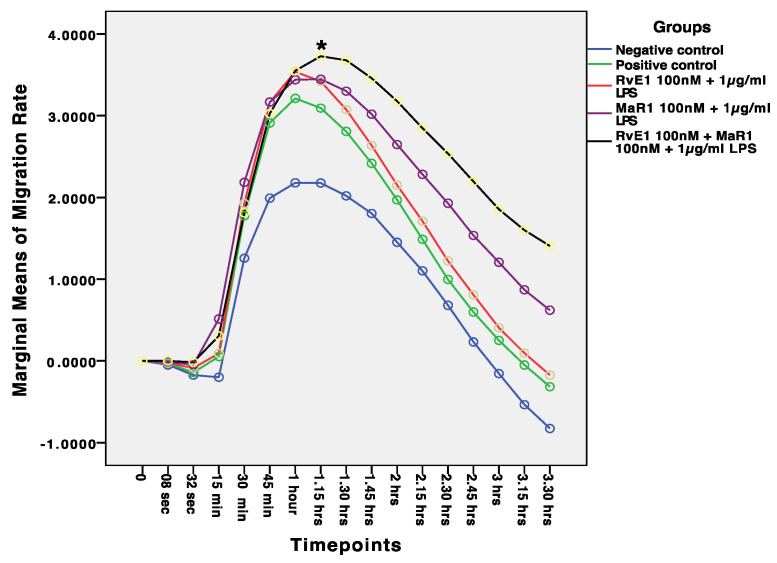
Comparison of mean migration values among the five study groups across different time points. The migration assays experiments were repeated three times (*n* = 3) and the mean value of migration of * Group-5 (RvE1 100 nM + MaR1 100 nM + 1 μg/mL LPS), was significantly higher than the other four study groups across 1 h to 3.30 h (*p* ≤ 0.0001).

The comparison of the mean migration among the five groups over 16 time points reveals a high statistical significant difference (*p* ≤ 0.0001). Additionally, there is statistical difference for the interaction term (Groups * Time points) (F = 3.49, *p* ≤ 0.0001). The average migration of the negative control was lower than the other study groups across nine time points (8 s to 1.45 h). Whereas no significant difference was noticed between Group 2–5 between 8 s to 1.45 h. Whereas the mean values of migration of Group-5 was statistically significant with higher mean values than the other four study groups across 1 h to 3.30 h (Figure 3).

### 3.4. Colony Forming Unit (CFU) Assay

**Figure 4:**
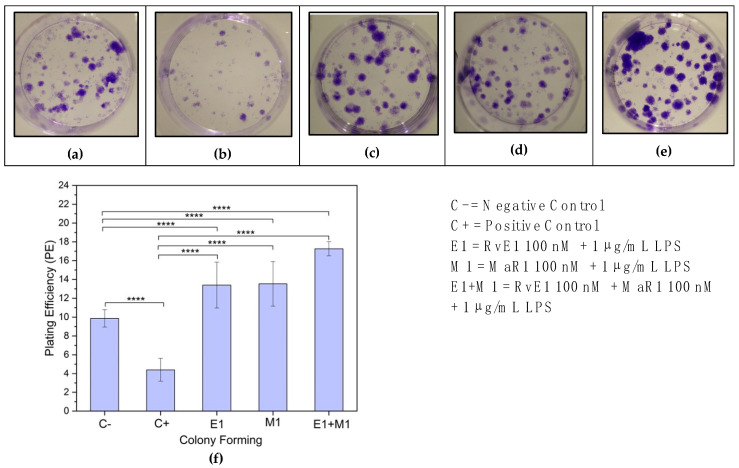
Clonogenic assay and plating efficiency at day 14 shows the mean values of CFUs among the five study groups, (**a**) Negative controls, (**b**) Positive controls, (**c**) RvE1 00 nM + 1 μg/mL LPS, (**d**) MaR1 100 nM + 1 μg/mL LPS and (**e**) RvE1 100 nM + MaR1 100 nM + 1 μg/mL LPS. Colony-forming unit (CFU) experiment was conducted three times (*n* = 3) for all experimental groups on three different plates simultaneously and average values calculated. Shown above is a representation of one of the stained plates (**a**–**e**) and quantitative histograms of averaged intensity representations. (**f**) CFU mean values of groups (3, 4, and 5) were significantly higher than controls as indicated in the figure. (**** *p* ≤ 0.0001).

The mean values of CFUs among the study groups showed high statistical significance with F = 24.38, *p* ≤ 0.0001. The multiple comparison test showed that the CFU of positive control (Group-2) was significantly less than the other four groups, whereas the mean CFU values of groups (3, 4 and 5) were significantly higher than the positive and negative control groups and no significant differences were noticed among these three groups. The following chart shows the comparison of mean values of CFUs among the five groups (Figure 4).

### 3.5. Western Blot of TGF-β1, IL-4, and IL-10

**Figure 5:**
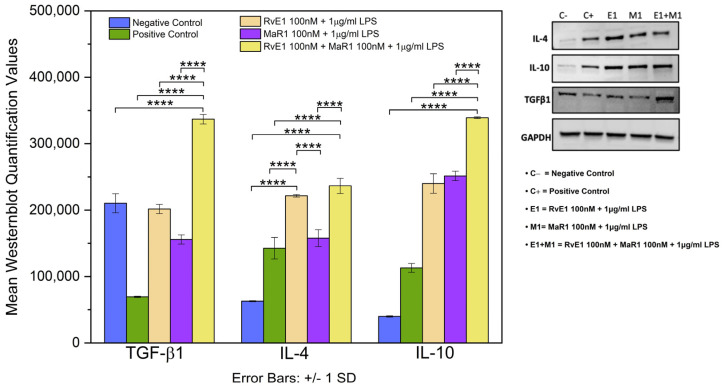
The right panel of the graph shows the expression of IL-4, 1L-10 and TGF-β1 between the five study groups and the left panel shown the normalized average values of three experimental repeats (*n* = 3). The expression of TGF-β1, IL4 and IL-10 was significantly higher in Group-5 (RvE1 100 nM + MaR1 100 nM + 1 μg/mL LPS) compared to the other four groups (**** *p* ≤ 0.0001).

Assessment of TGF-β1, IL-10, and IL-4 shows high statistical significant differences among the five study groups based on the normalized mean TGF-β1 (F = 268.772, *p* ≤ 0.0001), IL-4 (F = 85.446, *p* ≤ 0.0001), and IL-10 (F = 462.232, *p* ≤ 0.0001). The expression of TGF-β1 and IL-10 was significantly higher in Group-5 (RvE1 100 nM + MaR1 100 nM + 1 μg/mL LPS) when compared to the other four groups (Figure 5).

### 3.6. Western Blot Analysis of RANKL, TNF-α, and IFN-γ

**Figure 6:**
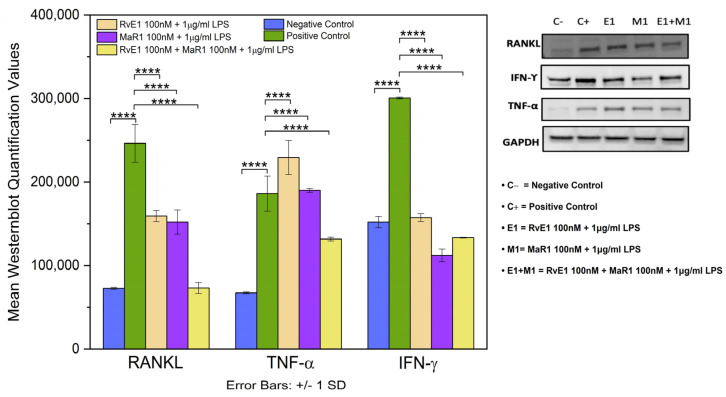
The right panel of the graph depicts the levels of protein expressions of RANKL, TNF-α, and IFN- γ by Western blot across the five study groups. Positive control has significantly higher RANKL and TNF- expression than the other four groups (Negative control, RvE1 + LPS, MaR1 + LPS, and RvE1 + MaR1 + LPS) (*p* ≤ 0.0001). Negative control group and Group 5 (RvE1+ MaR1 + LPS) have comparable levels of expression (*p* > 0.05). The left panel represents the normalized average values of three experimental repeats (*n* = 3) with their significant expressions. (**** *p* ≤ 0.0001).

Western blot analysis of RANKL, TNF-α, and IFN-γ revealed a high statistical significant difference in the normalized mean values among the five study groups, (F = 44.311, *p* ≤ 0.0001), (F = 412.596, *p* ≤ 0.0001), and (F = 64.303, *p* ≤ 0.0001), respectively. Among the five groups, the mean values of RANKL and TNF-α expression in Group-2 (positive control) were significantly higher than other four groups. The mean values of Group-1 (negative control) and Group-5 (RvE1 100 nM + MaR1 100 nM +1 μg/mL LPS) were significantly lower than those of the other three groups (Figure 6).

### 3.7. Human Cytokine Array

**Figure 7:**
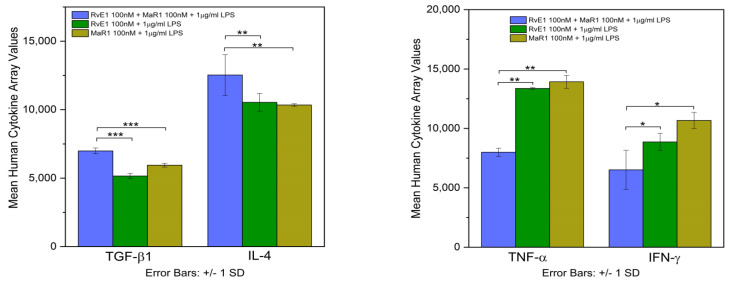
The left panel of the graphs show the expression of anti-inflammatory cytokines (IL-4 and TGF-β1), and the right panel pro-inflammatory cytokines (IFN-γ and TNF-α) by human cytokine array assessment in the three analyzed groups. The experiment was repeated three times (*n* = 3). (*** *p* ≤ 0.001, ** *p* ≤ 0.01, * *p* ≤ 0.05).

**Figure 8:**
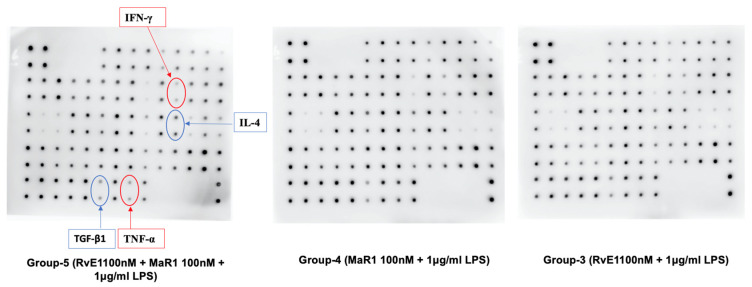
Human cytokines Antibody array membranes showing the expression of IL-4, TGF-β1, TNF-α, and IFN-γ among the three SPM treated study groups. Owing to limited supply of membranes for multiple repeats for all samples and groups, the triplicate runs (*n* = 3) were performed only for the experimental groups.

Human cytokine array assessment showed intensity of anti-inflammatory cytokines TGF-β1, and IL-4 were highly significant among the analyzed groups (F = 64.456, *p* = 0.001) and (F = 20.426, *p* = 0.007), respectively (Figure 7 and Figure 8). Briefly, among the three groups, the mean values of Group-5 (RvE1 100 nM + MaR1 100 nM +1 μg/mL LPS) were statistically significantly higher than Group-3 (RvE1 100 nM +1 μg/mL LPS) and Group-4 (MaR1 100 nM +1 μg/mL LPS) groups with no significant difference among these groups. Moreover, the pro-inflammatory cytokine expression of TNF-α and IFN-γ (Figure 7 and Figure 8) was shown to be highly statistically significant based on the mean values of human cytokine expression among the three study groups (F = 30.133, *p* = 0.003) and (F = 10.701, *p* = 0.022), respectively. The mean values of Group-5 (RvE1 100 nM + MaR1 100 nM +1 μg/mL LPS) were statistically significant with Group-3 and 4. However, no significance was noticed between Group-3 and 4.

### 3.8. Proteomics Enrichment Analysis and Characterization of Inflammatory Proteins

**Figure 9:**
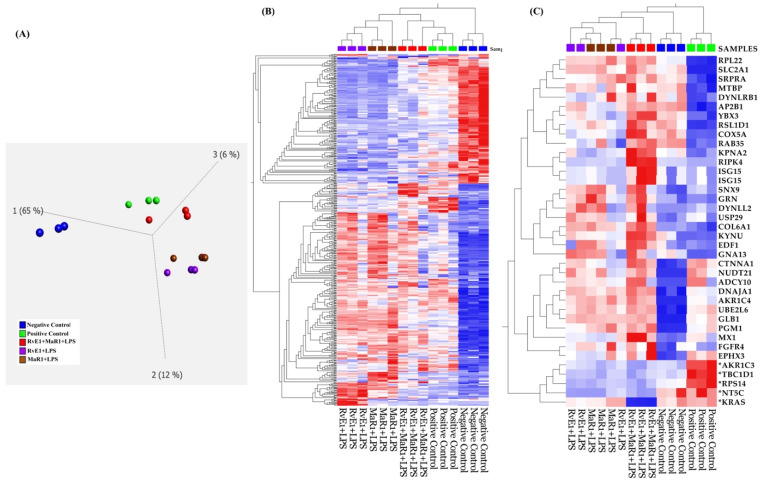
(**A**) Principal components analysis using the expression dataset of 464 differentially expressed proteins between Negative control, Positive control, RvE1 + LPS, MaR1 + LPS, and RvE1+ MaR1 + LPS. (**B**) Hierarchical cluster analysis using the expression dataset of the 464 differentially expressed proteins as described above. (**C**) Hierarchical cluster analysis using the expression dataset of 33 anti-inflammatory and 5 pro-inflammatory proteins with their expression changes. The heat maps show the expression changes of these proteins among the five sample groups. The figures were partly generated using Qlucore Omics Explorer version 3.7 (Lund, Sweden).

Whole cell lysates derived from hBMMSCs from each of the five groups were subjected to LC-MS proteomics expression. Over 1800 proteins were identified from all sample groups of which 464 were differentially expressed (>2-fold change, and *p* ≤ 0.05) (Supplementary Table S1). The 464 dataset was subjected to principal component (PCA) and hierarchical cluster analysis resulting in distinct sample clusters (Figure 9A,B).

**Figure 10:**
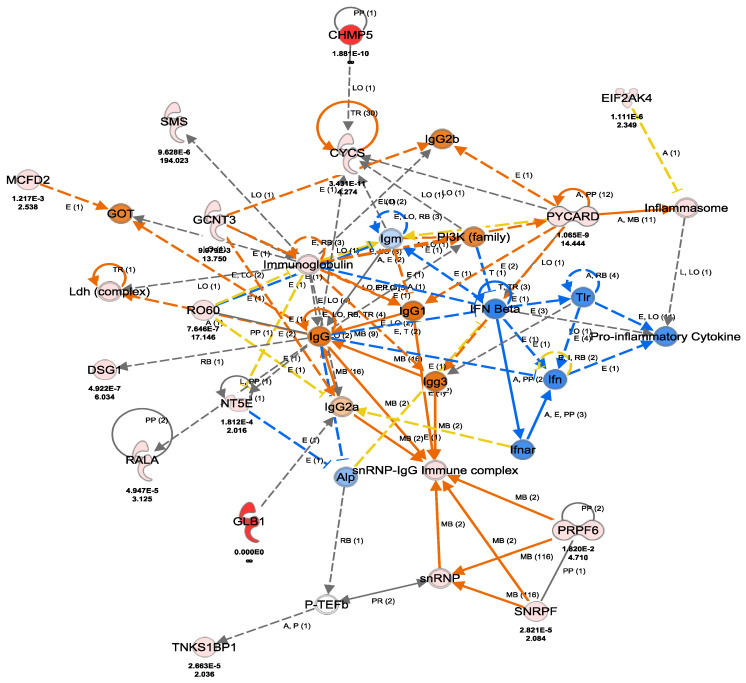
Ingenuity pathway analysis (IPA) of differential proteins linked with inflammatory response, immune complex and cytokines/inflammasome. The figure was generated using a licensed Ingenuity pathway analysis program (www.qiagen.com, accessed on 15 May 2022).

We further evaluated the 464 identified differentially expressed proteins among the five sample cohorts for their cellular processes, functional characteristics, and implications in immune-mediated or inflammatory disorders using ingenuity pathway analysis (IPA). The majority of the 464 proteins were associated with multiple signaling networks including cellular function and maintenance, metabolic disease, and organismal injury and abnormalities: immune response and inflammatory response. Others are cell death and survival, as well as cell-to-cell signaling and interaction. Of particular relevance to this study are 33 identified proteins that were involved in inflammatory response including pro-inflammatory cytokines and anti-inflammatory cytokines. The protein–protein interactions and gene names and expression changes of some of these proteins are as illustrated in Figure 9C and Figure 10.

## 4. Discussion

For a long time, it was believed that inflammation could be eliminated simply by diluting inflammatory mediators, resulting in the restoration of normal tissue function. Once inflammation has been initiated, the local immunological reaction will begin to express specific cytokines and immune mediators, which can influence the biological behaviour of the cellular composition of the local tissues including stem cells and influence the immuno-inflammatory environment. Endogenous specialised lipid mediators are secreted to prevent recruitment of neutrophil and to resolve inflammation [19]. The discovery that neutrophils are recruited endogenously to influence the immuno-inflammatory setting led to this understanding. As a result, it is now clear that uncontrolled or excessive inflammation is linked to an imbalance between SPMs and pro-inflammatory mediators [20,21]. In addition, it has been confirmed that SPMs are capable of actively resolving inflammation using chromatography methods [22,23]. These SPMs are biosynthesised from vital fatty acids. Alternatively, acetylation of COX-2 can potentially be employed as a technique for producing SPMs [7,24]. Because of structural variations, SPMs were classified into four families: lipoxins (LXs), resolvins (Rvs), protectins (PD), and maresins (MaRs) [25]. These molecules have wider application. Particularly, SPMs enhance the biological effects of G protein-coupled receptors by acting on those receptors (GPCR). SPMs can operate in a manner that is either an agonist [26,27], an inhibitor [28], or an allosteric agonist [29] to those receptors that are presented by immune cells. This allows them to exercise their influence on the host response [26,27]. As a direct result of this, the recruitment of neutrophils is inhibited by SPMs, whereas the recruitment of non-phlogistic monocytes in the post capillary venule is facilitated. In order to inhibit the spread of infection and inflammation, macrophages will phagocyte apoptotic neutrophils and any microorganisms that are present at the sites of inflammation [20,25]. This is done to protect the host from further infection and inflammation.

It is noteworthy that recent research shows SPMs in both the mouse and human support host homeostasis after stimulation or excitation with *E. coli* [30]. Particularly, rat vagus nerve only produces PD, but human vagus produces RvD5, RvE1, and MaR1 [30]. When the human vagus is stimulated, levels of MaR1, RvE1, RvD5, RvD3, and RvD4 rise. Vagus in the mouse generates 15-epi-LXA4, RvD4, RvE1, and RvE3 [30]. Accordingly, through regulating the PD conjugate in tissue regeneration [31], vagotomy prolongs the resolution of self-limiting E. coli infection. RvE1 and MaR1 have a distinct chemical structure and modulate cell signalling pathways through their ability to activate specific receptors and mediators. Resolvins block the production of proinflammatory mediators and regulate leukocyte trafficking to sites of inflammation and neutrophil clearance from mucosal surfaces. In particular, they restrict migration and infiltration of polymorphonuclear leukocytes [32]. MaR1, on the other hand, is produced by the action of 12-lipoxygenase on macrophages, platelets, and polymorphonuclear cells (PMNs) [33]. MaR1 constitutes a novel family of bioactive lipid mediators and has been shown to exert anti-inflammatory activity in many cell types, including bronchial epithelial cells, neutrophils, and macrophages [34]. As far as the current studies demonstrate, maresin cellular actions are primarily to promote M2 macrophage polarization and phagocytosis, inhibit neutrophil infiltration, and induce Treg generation [35]. Combining these compounds with their different modes of action to enhance their ability to resolve inflammation may potentially lead to better therapeutic efficacy. These findings demonstrate how SPM immunoresolvent capabilities position them as a novel class of medications for infection eradication by encouraging microorganism clearance and so reducing the need for antibiotic treatment. However, when hBMMSCs are inflamed, the regulatory influence of utilising RvE1 or MaR1 separately or jointly is a workable alternative for periapical inflammatory lesion therapy. Herein, we evaluate the individual and combined effects of Resolvin E1 (RvE1) and Maresin 1 (MaR1) on hBMMSC viability, proliferation, migration, survival ability, and expression of cytokines related to inflammation.

Results from this current study support the hypothesis that MaR1 and RvE1 improved inflammation resolution by enhancing hBMMSC viability, proliferation, and migration through regulating anti-inflammatory and proinflammatory markers (Figure 1 and Figure 3). These results are consistent with previously reported studies that MaR1 and RvE1 both rescued the regenerative capability of human periodontal ligament stem cells (hPDLSCs) by up-regulating periodontal ligament markers [36]. Our assessments were based on the levels of cell proliferation and viability in each of the five groups that were analysed. Despite the fact, however, that the majority of the treatment groups resulted in increased cell proliferation, with the highest levels observed in Group-5, which consisted of RvE1 and MaR1 in combination when exposed to LPS treatment, a study by Miyata et al., 2021 demonstrated the positive effect of SMPs on proliferation and activation of lymphoid cells that regulate the innate type 2 inflammation producing cells in a mouse model [37]. On the other hand, examination of RvE1 and MaR1cell damage-induced apoptosis revealed no evidence of its involvement. Alternatively, SPMs were reported to participate in phagocytosis of cells that have undergone apoptosis [38]. Furthermore, when the migration mean values from each of the five study groups were compared to one another, there was a significant difference between all of the groups (F = 121.36, *p* 0.0001), with Group-5 (RvE1 100 nM + MaR1 100 nM +1 μg/mL LPS) having the highest mean migration rate of cells. The other four groups had lower mean migration rates. Cell migration capacity is the primary necessary process that determines stem cell kinetics efficacy, and it suggests the use of highly migratory stem cells in cell-based therapies. Our results suggest the use of RvE1 100 nM + MaR1 100 nM that highly support the migratory efficiency of stem cells that can be beneficial in cell-based therapies, by improving stem cell homing to the injured area and subsequently increasing and stabilising therapeutic efficacy. Additionally, the ability of hBMMSCs to form colonies was assessed to examine the pattern of proliferation and differentiation. Among the analysed treatment groups, RvE1 and MaR1 together were noticed to be superior in the formation of colonies that were established in clonogenic assay. This pattern of SPM leverage results demonstrates the migration and clonogenic assays are consistent with a previous reported finding that RvE1 (100 nM) formed the highest number of colony units in comparison with other tested concentrations and accelerated the migration in an inflammatory inured in vivo model [16]. Considering the efficiency of cellular and molecular aspects of the treated hBMMSCs, the molecular behaviours were established based on Western blot, cytokine array, and LC/MS analysis.

TGF-β1, IL-10, IL-4 were significantly increased in Group-5 compared to the positive control Group-2 in the Western blot, while IFN-γ, RANKL, and TNF-α were decreased. Additionally, protein array validated the similar changes in TGF-β1, IL-4, TNF-α and IFN-γ in the expression of the proteins in the Group-5 condition. Changes in the expression indicate altered pro- and anti-inflammatory cytokine expression that was well established in SPMs. For instance, Serhan et al. report the pro-resolving lipid mediators’ ability to regulate a variety of molecules including pro- and anti-inflammatory molecules [34]. In addition, Croasdell et al. report that SPMs stimulate macrophages to produce IL-10 and TGF-β1 anti-inflammatory cytokines [39]. Alternatively, a study reports that resolvins could be better inhibitors of TNF-α and interleukin productions [40]. Additionally, our label-free quantitative liquid chromatographic mass spectrometry coupled with bioinformatics analysis provided the implicated proteomic profile across each condition and confirmed the potential immunological pathways regulating inflammatory response, pro-inflammatory cytokines and anti-inflammatory cytokines that contribute to the SPM treatment in an inflamed in vitro model.

In this study, RvE1 and MaR1 treatment significantly enhanced the anti-inflammatory microenvironment while suppressing the pro-inflammatory events. However, further research is needed to explain the various mechanisms involved in this resolution process by analysing the cellular and molecular signalling pathways in in vivo and in vitro experimental models.

Within the limits of our study, a growing body of this experimental evidence represents the first direct demonstration of the synergistic effects of RvE1 and MaR1 combination on hBMMSCs in regulating the resolution of the inflammatory response. This implies that when these mediators are dysregulated, inflammation may become excessive and persistent. Therefore, it will be of significant interest in the study of exogenous application of these endogenous mediators as intra-canal medicament agents in preventing periapical lesions from progressing into the irreversible stage and rescuing the unstable microenvironment.

## 5. Conclusions

Inflammatory response is a defense mechanism against injury or infection. Excessive or uncontrolled inflammatory reactions can lead to chronic diseases. The primary goal of this in vitro study was to provide proof that SPMs have a synergistic effect in resolving induced inflammation on hBMMSCs. Combination of SPMs may offer a novel therapeutic endodontic pharmacological approach to counteract the bacterial infection in pulpal-periapical pathosis. Resolvin E1 (RvE1) and Maresin 1 (MaR1) inhibited excessive inflammation while causing no significant cellular damage, thus combination of Resolvin E1 (RvE1) and Maresin 1 (MaR1) could be beneficial in the treatment as well as a promising therapy in maintaining hBMMSC vitality under inflammatory conditions.

## Data Availability

Details are presented within the paper in the Results and Supplementary Material sections. Additional data will be made available upon request from the corresponding author.

## Supplementary Materials

The following supporting information can be downloaded at: https://www.mdpi.com/article/10.3390/cells12010122/s1. Table S1: The 464 proteins expression dataset.
